# Chaotic Manifold Analysis of Four-Screw Extruders Based on Lagrangian Coherent Structures

**DOI:** 10.3390/ma11112272

**Published:** 2018-11-14

**Authors:** Xiang Zhe Zhu, Ying Tong, Yue Xin Hu

**Affiliations:** 1 School of Mechanical Engineering, Liaoning Shihua University, Fushun 113001, China; lshtongying@163.com; 2 School of Chemistry and Materials Science, Liaoning Shihua University, Fushun 113001, China; yxhu1981@163.com

**Keywords:** extrusion, four-screw extruder, finite-time Lyapunov exponents (FTLE), Poincaré section, chaotic manifold

## Abstract

The four-screw extruder (FSE) is a novel equipment for polymer processing. In this paper, from a new viewpoint of Lagrangian coherent structures (LCS), two-dimensional fluid transport and chaotic mixing characteristics within three kinds of novel industrial FSEs are explored based on LCS to better understand the flow and mixing natures in the FSEs. Firstly, based on the finite-time invariant manifold theory, the finite-time Lyapunov exponent (FTLE) and LCS of FSEs are calculated by considering the different initial time. Hyperbolic LCSs from the FTLE maps are adopted to identify chaotic mixing manifolds in FSEs. Moreover, particle tracking and Poincaré sections are used to illustrate the different fluid motions in the above three isolated regions. Finally, the effects of relative rotating directions and layout of four screws on the chaotic manifolds in FESs are discussed in order to enhance local mixing performance. Furthermore, quantitative mixing measures, such as the segregation scale, logarithmic of stretching, and mean-time mixing efficiency are employed to compare the mixing efficiencies in three kinds of FSEs. The results show that the relative rotating directions and positions of four screws can change the chaotic manifolds and increase mixing performance in local poor mixing regions. FTLE and LCS analysis are helpful to better understand the chaotic mixing nature in the novel screw extruders.

## 1. Introduction

As a classic piece of mixing equipment, single-screw and twin-screw extruders are widely used in the polymer-processing industry [1]. With the development of the polymer industry, some novel screw mixing elements in screw extruders, such as the pin mixing section [2], pitched-tip kneading disk [3], and screw profile with slots [4], are devised to obtain highly efficient mixing and a fine final product. Recently, a novel multiple-screw extruder, the four-screw extruder, was developed and has attracted more interest due to its many advantages, such as longer residence-time distribution, high output, great shear rate, and mixing efficiency [5]. The four screws of the typical four-screw extruder constitute a square arrangement resulting in four intermeshing regions and one special central region. However, the traditional twin-screw extruder only has two intermeshing regions and without central region. Due to the complicated geometry, the flow and mixing mechanisms in the four-screw extruder are very complex in comparison with the typical twin-screw extruder. Therefore, it is necessary to study the effects of four-screw extruder’s geometry, screw rotating manner on the flow, and the nature of the mixing in the four-screw extruder. More importantly, as a dynamic flow system, a fundamental understanding of the flow and mixing mechanisms in the extruders is helpful in controlling the desired flow for optimizing the geometrical structures and process conditions to guarantee the homogeneity of final product, which is also a primary aim of this article.

The key mixing mechanisms of laminar flow in screw extruders include dispersive and distributive mixing from a Eulerian viewpoint. In general, most of the studies have focused on the dispersive and distributive mixing to understand the mixing mechanisms in single- and twin-screw extruders [6,7,8,9,10,11,12]. Typically, Connelly et al. [10] adopted the segregation scale and cluster-distribution index to evaluate the distributive mixing in a 2D twin mixer. Moreover, the mixing index and shear stress were used to evaluate the dispersive mixing in the mixer. Domingues et al. [11] predicted the dispersive mixing performance from the extent deformation of the fluid drops, and mixing capability was quantified by the maximum line-stretching rate and the strain-rate-type identifier. Zhang et al. [12] adopted residence time distributions (RTD) to evaluate the distributive mixing in twin-screw mixers by particle tracking. Based on numerical and experimental investigations, many efforts were made to understand the relationship between the phenomena of the particle’s global behavior and the mixing process [13,14,15]. However, it is difficult to reveal the potential relationship between the chaotic manifolds and fluid transport in the mixers using the traditional Eulerian method.

Many studies have proved that chaotic mixing is an effective method to enhance mixing efficiency in the laminar flow of polymer processing in order to conquer the nature of laminar flow [16,17,18,19]. The Poincaré section and Lyapunov exponent (LE) are often carried out as general parameters to describe the chaos in a dynamical system. Lee et al. [18] suggested exponent stretch rate based on LE as chaotic mixing measures in single-screw extruder. The hyperbolic fixed point and chaotic manifold were obtained from the Poincaré map in the perturbed system. Hwang et al. [19,20] studied the evolution of chaotic mixing in the chaos screw (CS) nonlinear dynamical model using a Poincaré map based on a fourth-order Runge–Kutta scheme. The changes from the homoclinic fixed point and elliptic rotations to the resonance bands or KAM tori were analyzed depending on the commensurability of frequency ratio of the corresponding orbits. Niu [21] used the Poincaré section to locate the KAM curves and quasiperiod areas. The chaotic behavior of a dynamic system was evaluated by the Lyapunov exponent. Wang et al. [22] calculated the measure of the Kolmogorov–Sinai entropy rate from LE to discover the homogeneity of the system.

Recently, as a new measurement of chaotic mixing, Lagrangian coherent structures (LCS) were proposed to identify the chaotic manifold [23,24,25] in a dynamic flow system. LCS provide an effective tool to capture the potential dynamical features of a flow system, which could be missed in a traditional Eulerian analysis based on the velocity or vorticity field. The ridges of the finite-time Lyapunov exponent (FTLE) present the most stretching and repelling structures, which are called LCS (It is noted that several studies reported that the ridges of the FTLEs are not LCSs, as they have nonzero flux across them [25]). Many researchers used the FTLE and LCS to study fluid mixing and the transport process in internal mixers [26,27,28,29] and identify the vortex pinch-off [30]. Santitissadeekorn et al. [26] used the LCS to investigate the transport behavior and mixing process in a batch mixer. Robinson et al. [27] extracted the manifold structures from the forward and backward FTLE with a rational integration time, and the mixing characteristics in different conditions were discussed by using the LCS. Moreover, they used the same method to identify and visualize the 3D manifold intersections in a helical ribbon mixer [28]. In addition, Conti and Badin proposed a new method based on Covariant Lyapunov Vectors to describe hyperbolic patterns in two-dimensional flows [29]. Because the ridges of FTLE are equal to the boundary of transportation, the LCS were also widely used in other subjects, such as the atmosphere [31], oceans [32,33], biology [34], and electromagnetism [35]. However, the studies of using the LCS to analyze time-varying flow in polymer processing considering the moving parts are relatively limited.

As a novel extrusion device, four-screw extruders share distinct fluid-transport and mixing mechanisms in comparison to traditional twin-screw extruders. This paper explores the chaotic mixing and fluid-transport characteristics within three kinds of novel industrial four-screw extruders by employing FTLE, LCSs, and Poincaré sections. We focus on the two-dimensional chaotic advection and mixing in four-screw extruders using computational fluid dynamics. The chaotic manifold structures of the four-screw extruders were visualized by extracting the hyperbolic LCSs from the FTLE maps. Moreover, the Poincaré sections are used to assist in explore the mixing process in extruders and how material transport characteristics respond to changes in LCSs. Furthermore, quantitative mixing measures by means of the segregation scale, logarithmic of stretching. and mean-time mixing efficiency are employed to provide a rigorous method for comparison three kinds of four-screw extruders. The primary aim of this article is to better understand the nature and inherent of flow and mixing natures in the four-screw extruder.

## 2. Materials and Methods

### 2.1. Government Equations

In this study, 2D non-Newtonian and transient flow conditions are employed by using the finite-element method (FEM). The forms of continuity and momentum equations can be expressed as follows [36]:

(1) ∇⋅v=0

(2) −∇p+∇τ=v⋅∇v

where *p* is the pressure, τ is the extrastress tensor, and v is the velocity vector.

The stress tensor is given as:

(3) $$\tau =2\eta (\dot{\gamma })D$$

In which *η* is the viscosity, $\dot{\gamma }$ is the shear rate, and D is the rate-of-deformation tensor.

In this study, HDPE is processed in the four-screw extruders. It is appropriate to use the Carreau–Yasuda model to describe its rheological behavior as follows:

(4) $${\eta \;=\;\eta }_{\infty }\;+\;\left(\eta_{0}-\eta_{\infty }\right){\left[1\;+\;{\left(\lambda \dot{\gamma }\right)}^{a}\right]}^{{}^{\frac{n-1}{a}}}$$

where *η* is the dynamic viscosity, $\eta_{\infty }$ is the infinite shear viscosity, *λ* is a model-specific relaxation time, and $\lambda =\sqrt{2\prod_{D}}$. Π_D_ is the second invariant of the rate of deformation tensor and *n* is the power-law index. In this study, the material parameters of the HDPE melts at 200 °C are as follows: $\eta_{\infty }$ = 0 Pa·s, *η*_0_ = 113088 Pa·s, *λ* = 3.11 s, *n* = 0.36, *a* = 0.26.

### 2.2. FTLE and LCS

The Lagrangian description is considered as a better method to understand the idiosyncrasies of steady fluid flow. The LCS was used to describe the coherent structures of two-dimensional turbulent flow that defined as manifolds upon the dynamics. These manifolds are useful to understand the results of material transport from experimental and numerical flow data [26], and especially to explain the underlying mixing reasons of the high-viscosity fluid flow resulting in laminar flow.

The two-dimension flow dynamical system of four-screw extruders, the fluid point trajectory satisfied as:

(5) $$\{\begin{array}{c} \dot{x}(t)=v(x(t),t)\\ x(t_{0})=x_{0} \end{array}$$

where *x* is a fluid point trajectory on an arbitrary interval of time [*t*_0_, *t*]. The solution of the dynamical system given in Equation (5) in a certain time as a flow map can be viewed as a flow map. It is denoted by $\varphi_{t_{0}}^{t}$ and satisfies as follows [37]:

(6) $$\varphi_{t_{0}}^{t}:D\to D:x_{0}\mapsto \varphi_{t_{0}}^{t}(x_{0})=x(t;t_{0},x_{0})$$

Then, a finite-time version of Cauchy–Green deformation tensor by displacement grads tensor that form the trajectory *x*(*t*) of the dynamical system is obtained by

(7) $$C={\frac{{d\varphi }_{t_{0}}^{t_{0}+T}(x)}{dx}}^{*}\frac{{d\varphi }_{t_{0}}^{t_{0}+T}(x)}{dx}$$

where M* denotes the adjoint of M. *C* is the Cauchy–Green deformation tensor. The maximum and minimum eigenvectors of the *C* in Equation (7) imply that there are compression and expansion along the trajectory, respectively; and the maximum stretching occurs in direction aligned with the eigenvector associated with the maximum eigenvalue of *C*.

So, the FTLE with a finite integral time T can be defined as:

(8) $$\sigma_{t_{0}}^{T}(x)=\frac{1}{\left|T\right|}\ln\sqrt{\lambda_{\max}(C)}$$

In which, $\sigma_{t_{0}}^{T}(x)$ denotes the FTLE; *T* is associated to point *x*∈D at time *t*_0_; *λ*_max_(*C*) is the maximum eigenvalue of *C*. The LCSs are approximately obtained by the ridge of the FTLE field at time *t* for the initial position [37] and represent the stable and unstable material lines in the unsteady fluid flow. It may clearly understand the mixing and transportation behaviors in the unsteady flow. So, the LCS is a useful tool to quantify mixing in various notions.

For a periodic flow described in this paper, the LCSs corresponds to the hyperbolic invariant manifolds. The geometry of the hyperbolic manifolds can be found by calculating the spatial distribution of the finite-time Lyapunov exponent (FTLE) [23,24,25,26]. A repelling LCS normally appear in this field as a maximum ridge in the forward time FTLE field. Similarly, attracting LCS produce a maximum ridge in the backward time FTLE field.

### 2.3. Configurations of Three Types of Four-Screw Extruders

**Figure 1:**
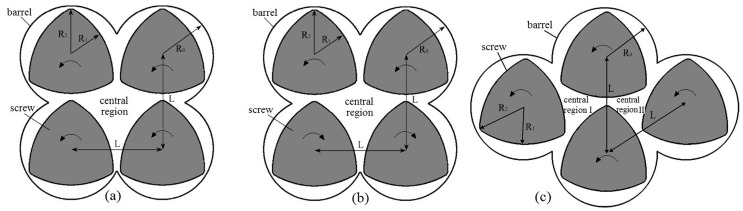
Three types of four-screw extruders at cross sections: (**a**) basic system (BS); (**b**) counter-rotating system (CRS); (**c**) screw equidistance distribute system (SEDS).

Based on the typical FSE, we developed two other types of new configurations. Therefore, four-screw extruders mainly involve three types geometries according to the arranged forms and rotating directions of the four screws, as follows: (i) The basic system (BS) is the corotating square-arranged four-screw extruder [5], in which the four screws arrange in square and all corotate counterclockwise, as shown in Figure 1a. (ii) The counter-rotating system (CRS), called a counter-rotating square-arrayed four-screw extruder, has four screws arranged in a square and the two top screws rotate counterclockwise, but the two bottom screws rotate clockwise, as shown in Figure 1b. (iii) The screw equidistance distribute system (SEDS), called an equidistance arrayed four-screw extruder, has four screws arranged in a rhombus shape and all corotate counterclockwise, as shown in Figure 1c. Detailed configurations and model parameters of the three types of FSEs at cross-sections are shown in Figure 1 and Table 1.

In comparison with typical twin-screw extruders, the three types of four-screw extruders have more intermeshing regions than twin-screw extruders, as shown in Figure 1. In addition, as can be seen from Figure 1a,b, the two models of corotating and counter-rotating FSEs, namely, the BS and CRS, both have four intermeshing regions and one central region where four screws cannot sweep the extrusion material. With the four-screw rotation, the area of the central region continually changes from big to small. However, in comparison with the BS and CRS, the SEDS has five intermeshing regions and two central regions with a relatively small area, as shown in Figure 1c.

### 2.4. FE Models and Computational Details

**Figure 2:**
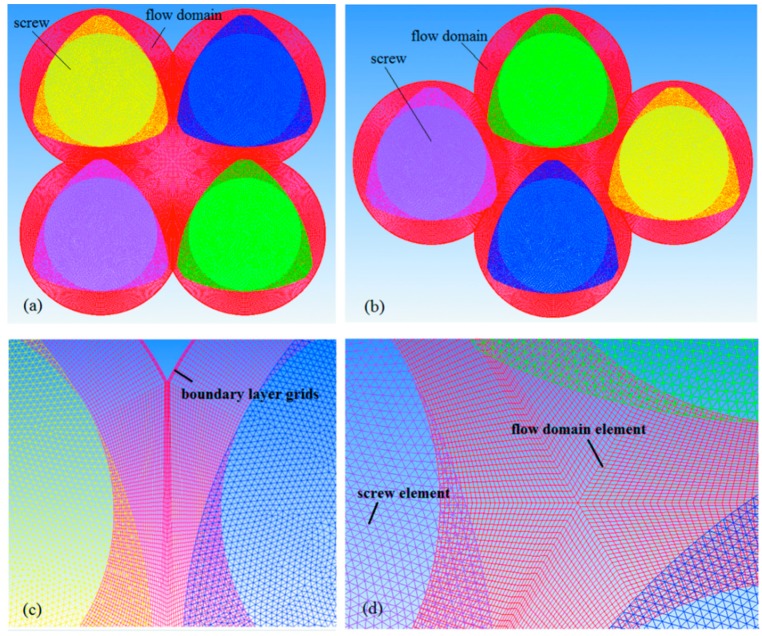
Finite-element models of the four-screw extruders in cross sections: (**a**) BS or CRS; (**b**) SEDS; (**c**) enlarged view of the gap region; (**d**) enlarged view of central region.

In this article, two-dimensional finite-element (FE) models without considering axial movement are employed to better understand the influences of geometric configurations on the flow-transportation and chaotic-mixing characteristics of four-screw extruders at cross sections. This is useful to achieve optimum shape design of cross section and improve mixing efficiency in practical applications. Three types of four-screw extruders were constructed using Solidworks software (2015, Dassault Systemes, Concord, MA, USA), as shown in Figure 1. Based on the mesh superposition technique (MST) [36], the FE models of the screws and barrel were established by using Gambit software (2.4.6, ANSYS Inc., Pittsburgh, PA, USA) without remeshing for periodical geometric changes. The quadrilateral and triangle elements were adopted to mesh the rotors and flow domain, respectively. In order to catch the small velocity changes in the small clearances between the rotors and walls and near the walls, four boundary layer grids in the FE model were employed, as shown in Figure 2. There is a total of 728,000 elements in the FE model of BS and CRS, respectively, and 637,800 elements in the FE model of SEDS. In order to ensure computational accuracy, more than 240 iterations were carried out per screw revolution. In our simulations, we chose a small-time step, *dt* = 0.5 s, which corresponds to 1.5° of screw rotation.

In this paper, two-dimensional flow fields in the mixer are calculated by using a commercial CFD code, ANSYS Polyflow, based on the generalized Newtonian approach. The convergence criterion was set to 1 × 10^−4^ in all the numerical cases. Moreover, based on the velocity fields in the extruders, the fluid-particle positions at time *t* + *T* were located by using the fourth-order Runge–Kutta scheme. Then, spatial gradient *dφ_t_x*(*t*)/*dx* was used to determine the Cauchy–Green deformation tensor for each initial point, and the FTLE map in the mixer was obtained at time t of each grid point from Equation (8).

### 2.5. Grid Independence and Time-Step Validations

**Figure 3:**
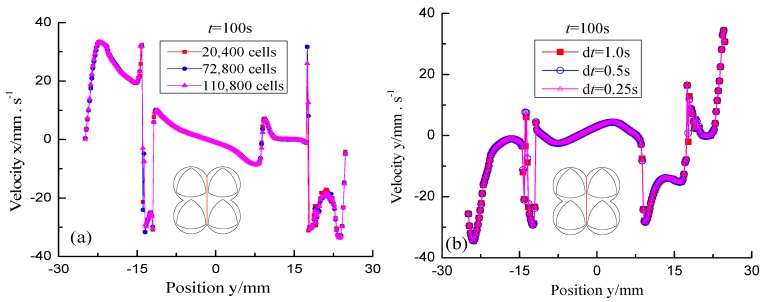
Effect of grid density and time step on the fluid velocity of the detected line: (**a**) Grid independence test; (**b**) time-step validation.

The effects of cell numbers in FE models on the numerical results is employed to validate the grid-independent [38]. Three kinds of FE models at different mesh interval sizes consist of 20,400 cells, 72,800 cells and 110,800 cells, respectively. The effects of grid elements on x-directional velocities of the red detected line across the screw channel are shown in Figure 3a. From this figure, it can be seen that the magnitudes of *x*-directional velocities in three kinds of FE models are almost same. Considering the computational cost, the FE model consisting of 72,800 cells was selected to accurately study the mixing mechanisms in the four-screw extruders.

To illustrate the effect of time steps on prediction, the red detected line was selected in the flow domain and was advected for 100 s using three different time steps, namely, d*t* = 1.0 s, d*t* = 0.5 s, and d*t* = 0.25 s, to detect velocity changing of the detected line in *y* direction, as shown in Figure 3b. This shows the trajectory agreement in detail. Time step d*t* = 0.5 s was chosen in our simulations considering the cost of computing.

## 3. Results and Discussion

### 3.1. Chaotic Manifolds in the Basic System

The Lagrangian method has high sensitivity to initial conditions. The spatial map of FTLE describes the dynamical evolution of each particle over interval time *T*. When the initial condition is stable, in principle, LCS positions are uniquely determined by their end positions. From a Lagrangian perspective quantifying a fluid-transport process, it is important to define the original condition and the integration time. After many calculations with different integration times, rotating revolution *T* is chosen as integration time for all simulations.

**Figure 4:**
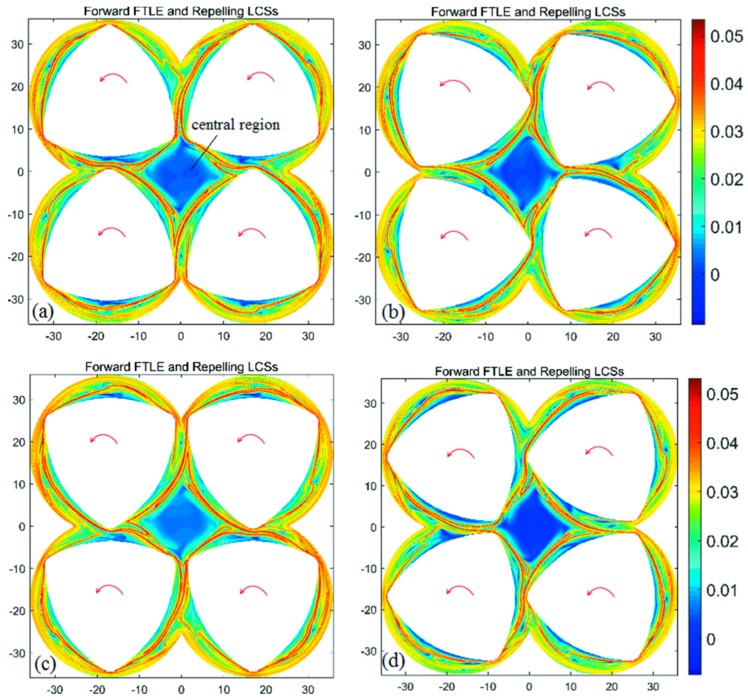
Forward-time finite-time Lyapunov exponent (FTLE) maps of the BS with integration time *T* = + a revolution and different initial time: (**a**) *t*_0_ = 0 s; (**b**) *t*_0_ = 10 s; (**c**) *t*_0_ = 20 s; (**d**) *t*_0_ = 30 s.

**Figure 5:**
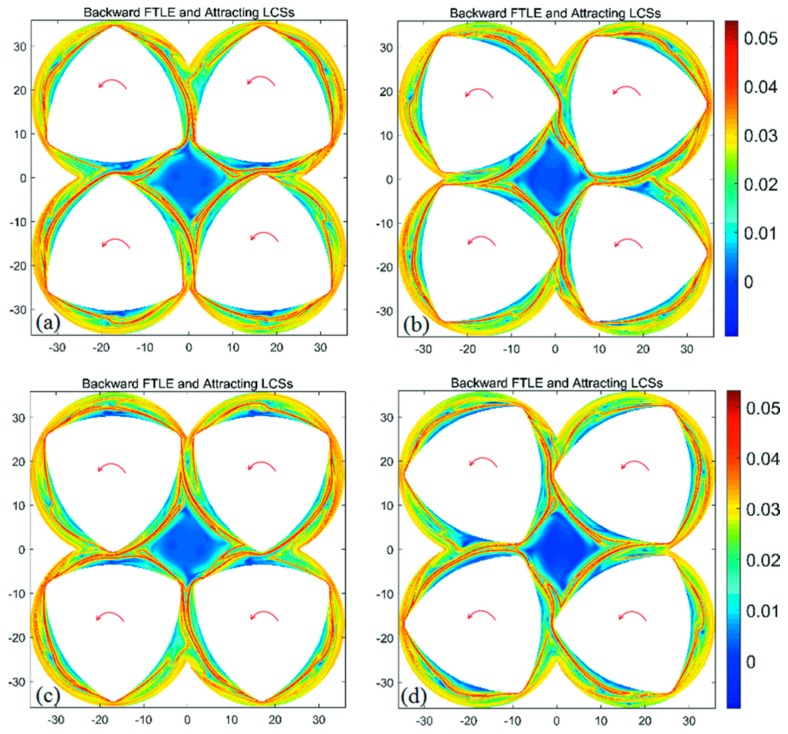
Backward-time FTLE maps of the BS with integration time *T* = −a revolution and different initial time: (**a**) *t*_0_ = 0 s; (**b**) *t*_0_ = 10 s; (**c**) *t*_0_ = 20 s; (**d**) *t*_0_ = 30 s.

The forward- and backward-time FTLE maps in the BS with integration time *T* = +a revolution and different initial time are shown in Figure 4 and Figure 5, respectively. It is clear from Figure 4 and Figure 5 that the maximal ridges in the FTLE maps show the location and change rule of repelling and attracting LCSs, and each screw is enclosed by the LCS. LCS form a closed diamond in the central region of the flow domain. Those fluid particles trapped in the central region are unable to mix with the rest of the flow domain. In addition, for all configurations of FTLE maps, LCSs slightly develop with the increase of initial time corresponding to different BS geometries. This leads to the similar diamond LCS structure in the central region, implying similar dynamic characteristics.

**Figure 6:**
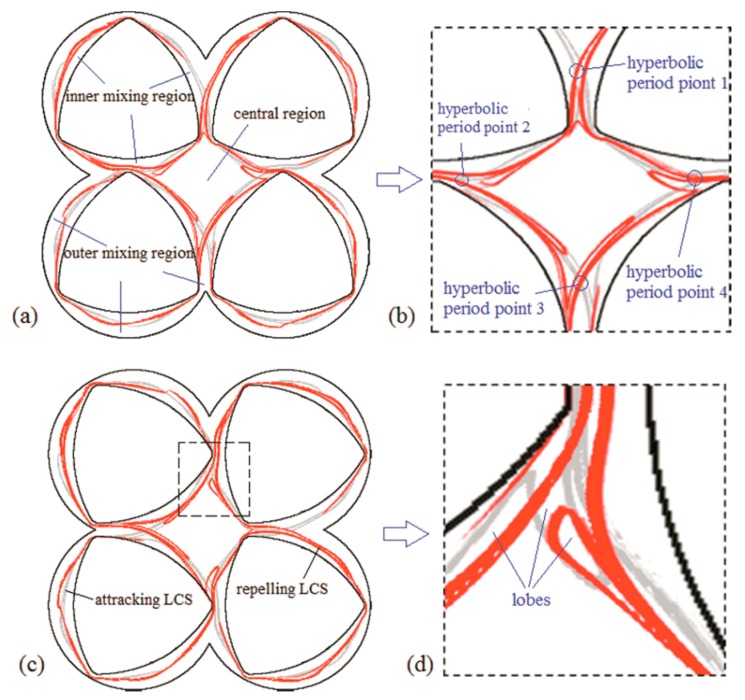
Repelling and attracting Lagrangian coherent structures (LCSs) redrawn from the FTLE maps with different initial time: (**a**) hyperbolic LCSs with initial time *t*_0_ = 0 s; (**b**) enlarged view of central region; (**c**) hyperbolic LCSs with initial time *t*_0_ = 10 s; (**d**) enlarged view of intermeshing region.

To locate the position of the hyperbolic fixed point in the flow system, the main repelling and attracting LCSs, namely, hyperbolic LCSs, in the BS with initial time *t*_0_ = 0 s and *t*_0_ = 10 s were redrawn, as shown in the Figure 6. It is clear from Figure 6 that there are four intersection points of hyperbolic LCSs, which are called hyperbolic periodic points. These intersections of manifolds indicate the presence of chaotic orbits. Moreover, hyperbolic periodic points are the dominant sources of mixing in the flow system. The fluid groups approaching the hyperbolic point are tangentially stretched away from the stable manifold and are folded along the length of the unstable manifold. Therefore, these flow regions that exist the hyperbolic periodic points have better mixing efficiency.

In the central region of the BS, the repelling and attracting LCSs tangle each other and emerge closed diamond of LCS structures, whose vertex is near the hyperbolic points. When a pair of hyperbolic LCSs come closer to being coincident, the tangle becomes a material transport barrier. The regions surrounded by the tangle only allow a small flux of fluid across the boundary. Therefore, we consider the tangle as the boundary that separates the physical flow domain into three portions with different mixing characteristics, as shown in Figure 6a. The first portion, which is called the outer mixing region, is located between the barrel wall and the repelling LCS. The second portion is called the inner mixing region and is located between the repelling LCS and the screw wall. The third portion encircled by the diamond LCSs is called the central region. The coincident hyperbolic LCS is the material transport boundary. A pair of hyperbolic LCSs are connected to adjacent intersection points to form a lobe structure set, which is the bridge of the material transport between adjacent regions with different mixing characteristics. In the outer mixing region, the stretching of fluid particles is better than that in the inner mixing region, and the mixing of fluid in the central region is the worst. So, the FTLE as a novel measure has potential advantages to quantity-mixing efficiency.

**Figure 7:**
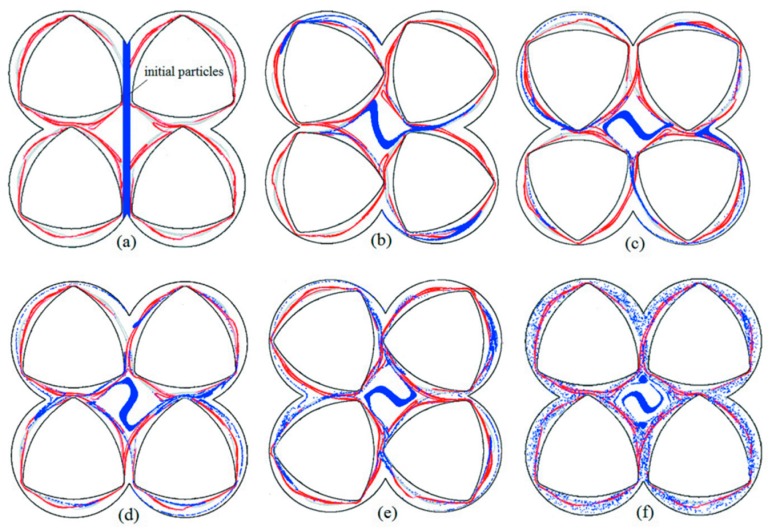
Particle-tracking results in the BS merging hyperbolic LCSs with different time: (**a**) *t* = 0 revolution; (**b**) *t* = 5/12 revolution; (**c**) *t* = 10/12 revolution; (**d**) *t* = 1 revolution; (**e**) *t* = 15/12 revolutions; (**f**) *t* = 5 revolutions.

To further understand the relative good or poor mixing characteristics in the three portions identified by the LCSs, particle tracking was used to illustrate the different fluid motions in the BS. Initially, we set free 3000 particles in a rectangle region located on the axis of the flow domain, which would pass through two hyperbolic periodic points, as shown in Figure 7a. Particle evolution over five revolutions in the BS is described in Figure 7b–f. It is noted that the simulations over five revolutions is sufficient to obtain a developed flow.

From the above analysis, we know that there are four hyperbolic periodic points at the center of the domain. Nearby, in every hyperbolic periodic fixed point, the particles would move apart faster. With the four screw rotations, the particle groups near hyperbolic periodic points 1 and 3 move along the unstable manifold into the left and right chambers, respectively, as shown in Figure 7b. The tangle also acts as the fluid-transport barrier; the attracting LCS indicate the mixing behaviors of tracer particles. In Figure 7c, the particles in the chamber (outer mixing region) are encircled by the barrel wall and hyperbolic LCSs, and only move with the screw rotation. Because those particles gradually move away from the hyperbolic periodic points, they are weakly stretched along the screw rotation. After a revolution, shown in Figure 7d, the particle tracers arrived at horizontal hyperbolic points 2 and 4. With the rotation of four screws, the particles affected by the horizontal hyperbolic points were further divided in opposite directions and entered different mixing characteristic regions. Then, the mixing process continued. From Figure 7f, it can be seen that hyperbolic LCSs overlap each other to form a series of lobe structures in the intermeshing region. Particles trapped in the outer mixing region identified by the LCSs employ material change with the inner mixing region by means of the lobe structures. The particle transports have perfect agreement with the boundary characteristics of the hyperbolic LCSs.

It is noted that the movement of those particles in the central region is limited by the transport boundary of LCSs. The closed-diamond LCSs mean that the tracers are substantially isolated and could not efficiently mix with the rest of the domain. Therefore, the dead zone in the central region formed by the closed LCSs must be considered to optimize the geometric deign and enhance the mixing efficiency of the BS. In the next section, in order to decrease or remove the dead zone, we study the effect of screw rotational direction and the arrayed manner of the four screws on the chaotic manifolds in four-screw extruders.

### 3.2. Chaotic Manifolds in CRS and SEDS

**Figure 8:**
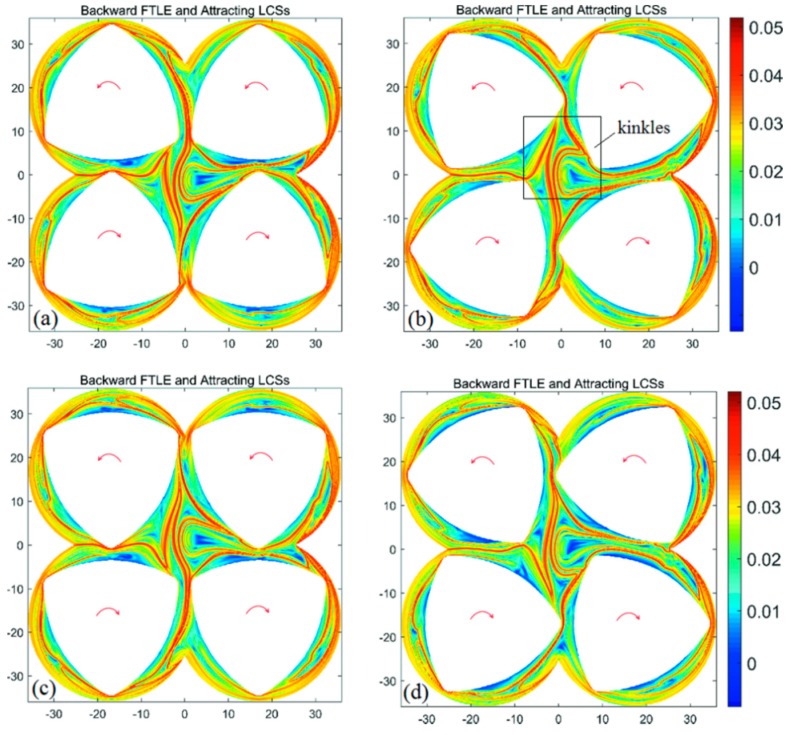
Backward-time FTLE maps in a CRS with integration time *T* = a revolution and different initial time: (**a**) *t*_0_ = 0 s; (**b**) *t*_0_ = 10 s; (**c**) *t*_0_ = 20 s; (**d**) *t*_0_ = 30 s.

Based on the base model of FSE, changing the direction of screw rotation is a relatively simple method to change the chaotic manifolds of a four-screw dynamic system. For the CRS, the two upper screws rotate counterclockwise, while the two screws below rotate clockwise. Figure 8 shows the backward-time FTLE maps in the CRS with a different initial time, where maximal ridges can correspond to the attracting LCS/unstable manifold. It can be seen from Figure 6 that the unstable manifolds in the CRS are complex in comparison with the BS. In the central region of the CRS, the closed diamond of the LCS structure disappears, and an open architecture of LCS structure appears. At the same time, many kinks appear in the CRS, implying the perfect folding action. It is known that the rate of fluid transport across the tangle of a manifold pair is proportional to the area of the lobes [27]. The kink structures in the CRS benefit from increasing the area of lobes, which is important to improve the fluid transport on both sides of the tangles. By changing the rotational direction of the screws, the closed fluid-transport boundary is destroyed to enhance local mixing efficiency in the central region and global mixing efficiency in the flow domain.

Based on the base model of BS, the relative positions of the four screws are varied to construct the SEDS model in order to obtain different flow domain from BS. For the SEDS, we explored the relationship between the four screw positions and the hyperbolic LCSs of the flow system, which are responsible for determining mixing performance.

**Figure 9:**
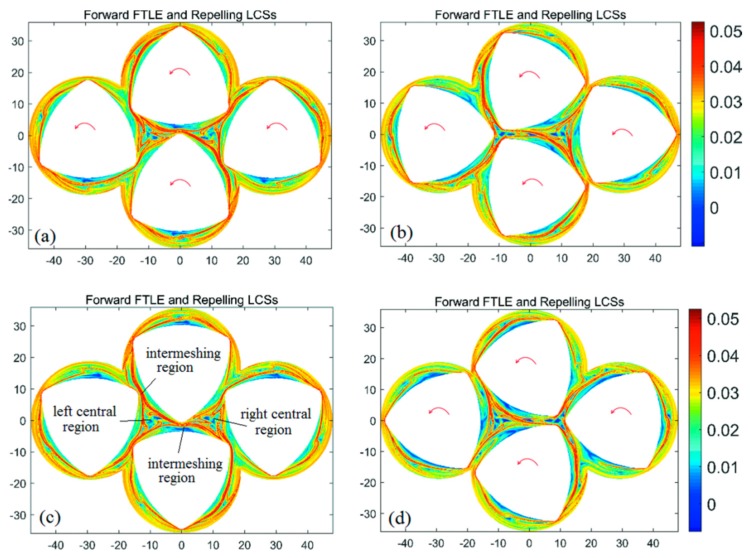
Forward-time FTLE maps in the SEDS with integration time *T* = a revolution and different initial time: (**a**) *t*_0_ = 0 s; (**b**) *t*_0_ = 10 s; (**c**) *t*_0_ = 20 s; (**d**) *t*_0_ = 30 s.

Figure 9 shows the forward-time FTLE maps in the SEDS using integration time *T* = 120 s. Ridges in these FTLE plots show the location of the repelling LCSs and wraps around the four screws. In particular, two small-area closed LCSs appear in the left and right central regions, respectively. With screw rotation, the area of the left central region decreases, corresponding to the decrease of the closed LCS areas, as shown in Figure 9b; however, the area of the right central region increases corresponding to the increase of the closed LCS areas. In comparison with the base BS model, there is only a relatively small blue zone in the two central regions of forward-time FTLE maps, implying better mixing efficiency than the BS. Moreover, the above periodic changes of the two central regions result in fluid in the periodic conditions of stretching and compression. This is beneficial to enhancing local mixing efficiency in the two central regions. More importantly, SEDS has five intermeshing regions corresponding to five hyperbolic fixed points, whereas the other two kinds of four-screw extruders only have four intermeshing regions. Due to the increase of the number of intermeshing regions, the mixing efficiency of the SEDS is improved to some degree. In addition, several kinks appear in the closed LCSs in the SEDS, implying perfect folding action of the fluid, and enhancing fluid exchange for both inward and outward of the closed LCSs.

### 3.3. Comparisons of Poincaré Sections

**Figure 10:**
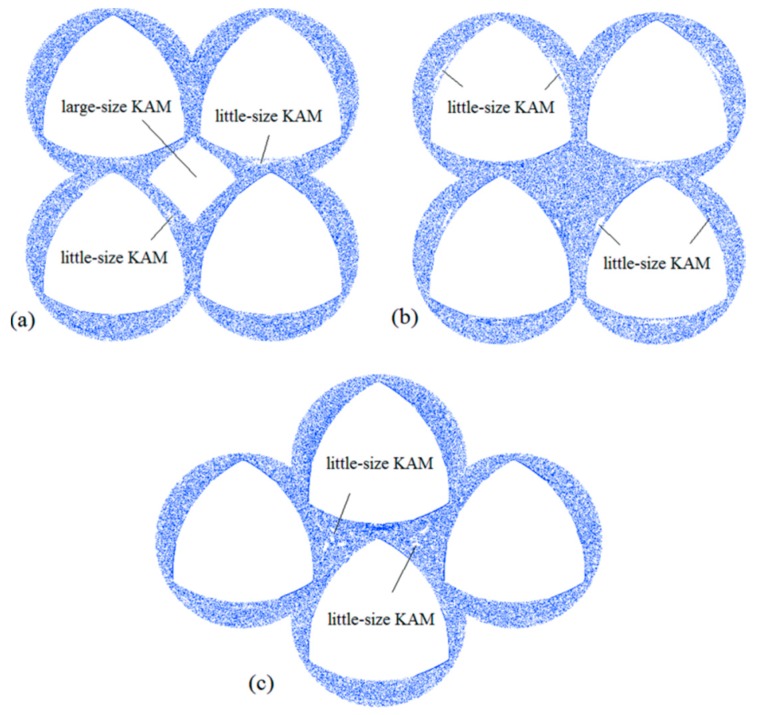
Comparisons of Poincaré sections of three types of FSEs: (**a**) BS; (**b**) CRS; (**c**) SEDS.

The Poincaré section is a simple method for analyzing chaotic mixing flows. It is a powerful way to reveal regular zones and chaotic motions. The Poincaré section allows for a systematic reduction in the complexity of problems by reducing the number of dimensions. Previously, many researchers have investigated the relationship between a Poincaré section and chaotic flow [39]. Here, we used the same method to plot the Poincaré section. Initially, 441 points were distributed in a box region with a size of 0.5 ×0.5 mm, and tracked their positions over 500 periods for obtaining the Poincaré sections of three kinds of models, as shown in Figure 10.

As shown in Figure 10a, there was a large-size KAM island that can correspond to the closed diamond repelling LCS in the central region of the BS. This indicates the periodic property in the KAM island in which particles hardly mix with other regions in the BS. They served as the main barrier to better mix in the corotating four-screw extruders. In addition, several little-size KAM islands appeared near the screw walls. This is associated with the screw geometry. In comparison with the BS, there was no KAM island in the central region of the CRS, and only several little-size KAM islands appeared near the screw walls, as shown in Figure 10b. This phenomenon can be explained by the above analysis of LCS structures. This is different from the BS and SEDS, as the SEDS has two relatively small central regions. As shown in Figure 10c, there were several little-size KAM islands that appeared in the two central regions. Around these KAM islands, the fluid in the rest of the flow domain was in chaos. From the comparisons of the Poincaré sections in four kinds of four-screw extruders, we can conclude that KAM regions in the BS are the largest, followed by the SEDS, and those in the CRS were smallest. Therefore, it is deduced that the CRS has maximal mixing efficiency, followed by the SEDS, and the BS has minimal mixing efficiency.

### 3.4. Quantitative Mixing Comparisons

**Figure 11:**
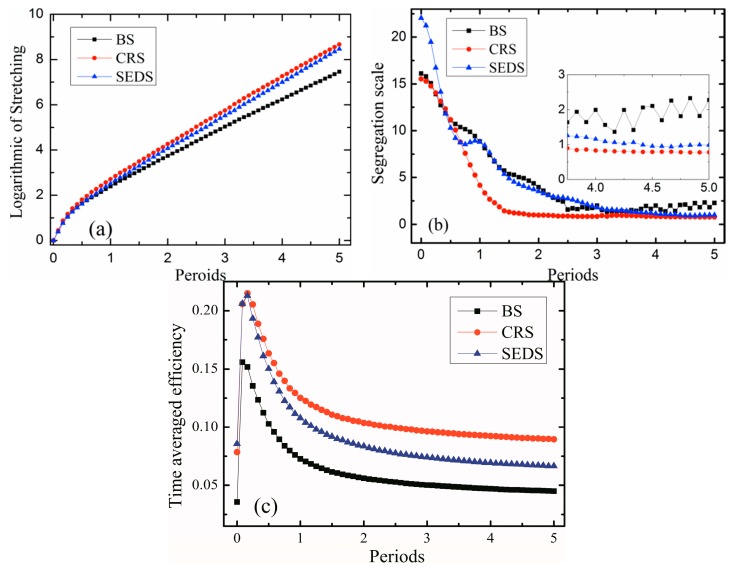
Comparisons of different mixing measurements between BS, SEDS, and SEDS: (**a**) logarithmic of stretching; (**b**) segregation scale; (**c**) time-average efficiency.

In order to further understand the influences of the screw rotational manner and geometric configurations of four-screw extruders on chaotic mixing, the logarithm of stretching, segregation scale, and time-averaged efficiency were calculated and compared with the three types of four-screw extruders. Initially, 10,000 massless particles were injected in the flow domain, and particle statistics were employed to obtain the above parameters over five periods, as shown in Figure 11a–c.

The logarithmic of stretching is often used to evaluate fluid stretching [10]. Given motion *x* = *χ*(*X*,*t*), where initially *x* = *χ*(*X*,*t*) for an infinitesimal material line segment *x* = *χ*(*X*,*t*), located at position *x* at time *t*, the length of stretch of a material line is defined as:

(9) $$\lambda =\frac{\left|dx\right|}{\left|dX\right|}$$

Figure 11a shows the plot of the logarithmic of stretching versus time for three kinds of four-screw extruders. The logarithmic of stretching in three models all increase exponentially over time due to the folding of the polymer melt between the four screws. The CRS showed better length of stretching, followed by SEDS, and then BS. This is due to the fact that the BS has a large-scale poor stretching mixing region in the central region, identified by the closed diamond LCSs, and the SEDS only has two little-area stretching mixing regions in the two central regions, but it has five intermeshing regions. The CRS, on the other hand, has no poor stretching mixing region in the central region.

The segregation scale is a measure of homogeneous concentration in flow regions [10] and can be expressed by:

(10) $$S(t)=\int_{0}^{\zeta }R\left(r,t\right)dr$$

where *R*(*r*,*t*) is the correlation coefficient for the concentration, and it gives the probability of finding a pair of random points with relative distance *r* with the same concentration [10]. The segregation scale is a measure of the size of the regions of homogeneous concentration, which decreases when mixing improves.

Figure 11b shows the segregation scale plotted over five revolutions for all three models. It can be seen that the three kinds of models showed a rapid drop during 1.5 periods (revolutions) due to the large segregated area. The CRS had a smaller segregation scale than the two other kinds of models, implying better distributive mixing efficiency due to no obvious mixing dead region. After 3.5 periods, the SEDS had a smaller segregation scale than the BS, because the SEDS has five intermeshing regions and a relatively small-sized central region. However, the relatively great magnitude of segregation scale in the BS had a little increase, from 3.5 to 5 revolutions, due to the main barrier of better mixing in the central region.

Time-average efficiency is often used to describe the stretching mixing efficiency during mixing. It is defined as:

(11) $$\langle e_{\lambda }\rangle =\frac{1}{t}\int_{0}^{t}\frac{\dot{\lambda }/\lambda }{{\left(D:D\right)}^{1/2}}dt$$

where $\langle e_{\lambda }\rangle$ is the time-average mixing efficiency, and D is the rate of strain tensor. It is clear from Figure 11c that the CRS showed better mean-time mixing efficiency, followed by the SEDS and BS. At five periods, the mean-time mixing efficiency of the CRS and SEDS was 2.2 times and 1.5 times of that of the BS, respectively. Therefore, to change the relative screw rotating direction and the positions of four screws can have great effects on the flow and mixing nature of four-screw extruders.

## 4. Conclusions

This paper explores the chaotic-mixing and fluid-transport characteristics within three kinds of novel industrial four-screw extruders by employing FTLE, LCSs, and Poincaré sections. We focused on two-dimensional chaotic advection and mixing in four-screw extruders using computational fluid dynamics. The manifold structures of the four-screw flow systems were visualized by extracting the hyperbolic LCSs from the FTLE maps. Hyperbolic LCSs as fluid-transport boundaries were used to analyze the material transport in two-dimension flow field of FSEs. Moreover, Poincaré sections were employed to indicate the chaotic and regular regions, which are key in improving local mixing. Furthermore, quantitative mixing measures by means of the segregation scale, logarithmic of stretching, and mean-time mixing efficiency were employed to provide a rigorous method for comparison of the three FSE systems.

In the BS, namely, the corotating square-arranged four-screw extruder, the hyperbolic LCSs have four hyperbolic fixed points in the four intermesh regions, which are important to enhance mixing efficiency due to the fluid stretching and folding actions, which can help us better understand the better mixing performances in the intermeshing regions. Hyperbolic LCSs, as an underlying transportation boundary, separate the fluid flow of the mixer into three isolated regions, namely, the inner mixing region, outer mixing region, and central region. In the outer mixing region, the fluid was stretched strongly, indicating better mixing efficiency. In the inner mixing region, however, mixing was relatively poor. However, the central region, surrounded by the closed diamond LCSs, was a dead region, in which there was a large-size KAM island in the Poincaré sections of the BS. This obviously decreases the global mixing efficiency in the BS. Correspondingly, the mixing-efficiency measurement of the segregation scale, logarithmic of stretching, and average-time mixing efficiency was lower than the other two FSE systems. So, the central region should be considered to optimize the manifold geometry controlled by the BS profiles to enhance mixing efficiency.

In the CRS, namely, the counter-rotating square-arrayed four-screw extruder, the geometry of the flow field was the same as that of the BS. However, the corotating and the counter-rotating manners of the four screws was relatively different from that of the BS. This caused the dead region in the central region of the flow domain to disappear from the analysis of the repelling LCS and Poincaré sections. Local mixing efficiency in the central region was obviously enhanced. Furthermore, the CRS had a more complex manifold structure and great numbers of kinks in the hyperbolic LCSs, resulting in stronger fluid stretching and transport. Therefore, the CRS had a smaller segregation scale, and greater logarithmic of stretching and average-time mixing efficiency than the other four-screw systems. So, the screw rotating manner has a great influence on the manifold structure and mixing efficiency in a four-screw flow system.

The SEDS, namely, the equidistance-arrayed four-screw extruder, has five intermeshing regions and two central regions, which are different from the BS and CRS. The manifold structure of the SEDS has five hyperbolic fixed points in five intermeshing regions, implying better local mixing performance. In addition, several kinks appeared in the closed LCSs in the SEDS, implying perfect folding action of the fluid, and enhancing fluid exchange for both inward and outward of the closed LCSs. Therefore, the SEDS had a smaller segregation scale, and greater logarithmic of stretching and average time mixing efficiency than the BS. However, two small-area closed LCSs appeared in the left and right central regions, which corresponded to several little-size KAM islands. This resulted in relatively poor mixing in the SEDS compared to the CRS, because the CRS had no dead region in the central region. So, varying the relative positions of screws could change the manifold structure and enhance the mixing efficiency of the four-screw flow system.

This paper has shown that a fresh Lagrangian perspective is more feasible than the traditional Eulerian method in numerically investigating the evolution of two-dimensional mixing performance within a novel screw extruder. FTLE and LCS are useful tools for analyzing chaotic mixing flow. This method is robust and flexible, and can be applied to other types of polymer-processing equipment. It provides a better understanding of the mixing mechanism in screw extruders.

## Figures and Tables

**Table 1:** Geometric parameters of four-screw extruders.

Parameter	Value
Barrel diameter R_0_	18.5 mm
Screw root diameter R_1_	13 mm
Screw tip diameter R_2_	17 mm
Centerline distance of four screws L	33 mm
Screw clearance	3 mm
Clearance of screw and barrel	1.5 mm
Rotational speed of four screws	0.5 r/min
Leads of our screws	3
